# Preventing chronic low back pain: investigating the role of Pilates in subacute management—a randomized controlled trial

**DOI:** 10.1007/s11845-025-03939-y

**Published:** 2025-04-04

**Authors:** Hatice Kubra Asik, Tugba Sahbaz

**Affiliations:** Faculty of Medicine, Department of Physical Medicine and Rehabilitation, Istanbul Beykent University, Istanbul, Turkey

**Keywords:** Exercise, Low back pain, Quality of life

## Abstract

**Background:**

Subacute low back pain (LBP) is a critical phase that can determine long-term patient outcomes. Exercise therapy, including Pilates, is widely used to manage LBP, but its effectiveness in the subacute phase remains underexplored. This randomized controlled trial aims to compare the effects of an 8-week Pilates-based intervention versus a home exercise program on pain intensity, functional disability, and quality of life in patients with subacute LBP.

**Methods:**

Sixty-six participants with subacute LBP were randomized into two groups: a supervised Pilates group and a home exercise group. Pain intensity (Visual Analog Scale), functional disability (Roland-Morris Disability Questionnaire), and quality of life (Short Form-36) were assessed at baseline, post-intervention, and 3-month follow-up.

**Results:**

Both groups exhibited significant improvements in all outcome measures (*p* < 0.001). However, the Pilates group showed superior reductions in pain intensity (*p* = 0.010 post-treatment, *p* = 0.002 at follow-up) and functional disability (*p* = 0.009 and *p* = 0.002). Additionally, quality of life scores improved more significantly in the Pilates group, particularly in physical function (*p* = 0.031 and *p* = 0.025) and general health (*p* = 0.005 and *p* = 0.012).

**Conclusions:**

Pilates-based rehabilitation was more effective than a home exercise program in improving pain, disability, and quality of life in patients with subacute LBP. These findings support the inclusion of Pilates in early-stage rehabilitation to prevent chronicity.

**Trial registration:**

Clinical Trials Number: NCT06699511.

## Introduction

Low back pain (LBP) is one of the most prevalent musculoskeletal disorders globally, with a lifetime prevalence of 84% and significant societal and economic burdens, including disability, absenteeism, and healthcare costs [1, 2]. It is the leading cause of years lived with disability worldwide, and its prevalence continues to rise due to population aging and other factors [3, 4]. LBP is often classified into acute, subacute, and chronic phases based on symptom duration. The subacute phase—lasting 4 to 12 weeks—plays a critical role in determining long-term outcomes. During this period, symptoms may either resolve or progress into chronicity, which affects 10–20% of patients and is associated with greater disability and healthcare costs [4, 5].

Exercise therapy is widely recognized as a cornerstone of LBP management due to its evidence-based benefits in alleviating pain, improving functional capacity, and enhancing quality of life [6, 7]. Among various exercise interventions, Pilates has emerged as a promising approach for addressing chronic LBP, focusing on core stabilization, postural alignment, and precise movement patterns [8]. Pilates emphasizes the activation of key stabilizing muscles such as the transversus abdominis and multifidus, which are essential for lumbar control and functional recovery [8, 9]. Meta-analyses and systematic reviews have demonstrated that Pilates significantly reduces pain and disability while improving physical function and postural alignment [10, 11]. Its adaptability for individualized interventions further enhances its appeal for diverse patient populations, including those in the subacute phase [12].

Despite the well-documented benefits of Pilates for chronic LBP, its application during the subacute phase remains underexplored. Clinical practice guidelines emphasize early, evidence-based interventions during this transitional period to prevent chronicity, reduce pain intensity, and restore function [13, 14]. Supervised Pilates programs, in particular, have been highlighted as effective in addressing muscle imbalances, promoting adherence, and enhancing motor control compared to traditional home-based exercise programs [14, 15]. However, there remains a significant gap in the literature regarding the comparative effectiveness of Pilates and other exercise modalities for subacute LBP patients.

In addition to exercise therapy, guidelines for subacute LBP management also recommend active treatments such as motor control and spinal stabilization exercises, which engage patients in their recovery process and promote long-term self-management skills [16]. Research further suggests that integrating core stabilization and motor control exercises with manual therapy techniques, such as spinal mobilization, can enhance patient outcomes [10]. These findings underscore the importance of evaluating Pilates as an independent and adjunctive intervention to optimize recovery during the subacute phase.

This study aims to address these gaps by comparing the effects of an 8-week Pilates program to a home exercise regimen in patients with subacute LBP. The primary outcomes include pain intensity, functional disability, and quality of life. By focusing on this underrepresented patient population, the findings will provide valuable insights into the role of Pilates as an early intervention strategy, potentially improving short-term recovery and reducing the long-term burden of chronic LBP.

## Materials and methods

### Study design

This study is a prospective, randomized, single-blind, controlled trial designed to evaluate the effects of an 8-week Pilates exercise program compared to a home exercise program in patients with subacute LBP. Participants were randomly assigned into two groups: the Pilates group and the home exercise group. Participants were randomized using a computer-generated sequence. The study took place between November 1, 2022, and July 1, 2024, at the Physical Medicine and Rehabilitation Outpatient Clinic of Istanbul Beykent University Hospital. While the physiotherapists and participants adhered to their respective exercise regimens, outcome measures were assessed by a blinded evaluator to minimize bias.

Participants included adults aged 18 to 65 years with a diagnosis of non-specific subacute LBP lasting between 6 and 12 weeks. Individuals with specific causes of LBP (e.g., herniated disc and spinal stenosis), previous spinal surgery, severe comorbidities, pregnancy, or conditions preventing safe exercise participation were excluded. The sample size estimation was designed to achieve at least 80% power to detect a clinically meaningful 2.5-point difference between groups in the primary outcome measure, the Roland Morris Disability Questionnaire. The calculation was performed using G*Power 3.1.9.2, assuming a common standard deviation of 3.7 points based on previous literature [17]. A two-group, one-tailed *t*-test with a significance level of 0.05 and 80% power determined that 28 participants per group were required. Accounting for a 15% anticipated drop-out rate, the final sample size was adjusted to 33 participants per group, resulting in a total of 66 participants.

### Interventions

Participants in the study were randomly assigned to either the Pilates group or the home exercise group, both of which followed an eight-week intervention program tailored to address subacute LBP.

The Pilates group attended supervised sessions three times per week, totaling 24 sessions over the study period. Each session, lasting approximately 60 min, was led by a certified physiotherapist with expertise in Pilates-based rehabilitation. The exercise protocol aimed to improve core stabilization, lumbar segmental control, and functional recovery, with intensity progressively increasing based on participants’ abilities. Sessions began with a warm-up phase that included gentle mobilization exercises such as pelvic tilts, Cat-Cow stretches, and spinal articulation movements to prepare the muscles and joints for activity [18]. The main workout focused on core stabilization and strength training, incorporating exercises such as the Hundred, Plank variations (forearm, side, and dynamic), Single-Leg Stretch, and Double-Leg Stretch, all of which target deep core muscle activation and enhance spinal stability [19]. Additionally, flexibility and functional movements like the Spine Twist, Side-Lying Leg Lifts, and Seated Forward Fold were included to improve lumbar range of motion and support daily activities [20]. The program followed a structured progression strategy, with modifications such as adding resistance bands, increasing repetitions, extending plank durations, and incorporating unstable surfaces like stability balls when appropriate. Each session concluded with a cool-down phase, featuring exercises such as Child’s Pose, gentle spinal rotations, and hamstring stretches to aid recovery and reduce post-exercise stiffness.

The home exercise group followed a structured, progressive program designed for independent practice without the need for specialized equipment. Participants performed general stretching, strengthening, and mobility exercises three times per week, with modifications introduced based on their progress. The program began with a warm-up phase that included pelvic tilts, Cat-Cow movements, and light spinal mobilization exercises to activate lumbar and abdominal muscles [21]. Strengthening exercises such as Glute Bridges, Bird Dog, Side-Lying Leg Lifts, and Modified Squats were incorporated to improve core and lower back strength, while mobility and flexibility training, including Trunk Rotations, Hamstring Stretches, and Quadriceps Stretches, aimed to alleviate stiffness and enhance functional mobility [9, 18]. To ensure gradual progression, participants were encouraged to increase repetitions, hold positions longer, or add light resistance, such as ankle weights or resistance bands, as tolerated. Adherence to the program was closely monitored; participants-maintained exercise logs and were contacted weekly by the research team to track compliance, address concerns, and provide guidance when necessary.

Both groups were carefully monitored throughout the study for adherence, progression, and any adverse events to ensure a safe and effective intervention.

### Outcome measures

The primary and secondary outcomes of the study were assessed at three time points: baseline (before the intervention), immediately after the eight-week program, and at a three-month follow-up. Pain intensity was evaluated using the Visual Analog Scale (VAS), which ranges from 0 to 10. Functional disability was assessed using the Roland-Morris Disability Questionnaire (RMDQ), a widely utilized instrument designed to measure the impact of LBP on physical functioning. The RMDQ consists of 24 items, each addressing specific limitations in daily activities due to back pain, such as walking, sitting, and performing household tasks. Higher scores indicate greater disability, reflecting a more substantial functional impairment [22]. This questionnaire has been extensively validated in various populations, including Turkish-speaking patients, ensuring its reliability and cultural adaptability for assessing disability in individuals with LBP [23].

Quality of life was assessed using the Short Form-36 (SF-36), a widely validated and comprehensive instrument designed to measure both physical and mental health. The SF-36 evaluates health-related quality of life across eight domains, including physical functioning, bodily pain, general health perceptions, social functioning, vitality, emotional role limitations, physical role limitations, and mental health. Higher scores indicate better health-related quality of life [24]. This instrument has been validated for use in Turkish-speaking populations, ensuring its reliability and applicability in assessing quality of life in individuals with musculoskeletal conditions [25]. Adherence to the intervention protocols was closely monitored through weekly check-ins and exercise logs. Any adverse events occurring during the study period were documented and managed appropriately. This comprehensive approach to outcome measurement ensured a thorough evaluation of the interventions’ effectiveness. Assessments were performed at baseline, immediately post-intervention (8 weeks), and at 3-month follow-up.

### Ethical considerations

The study was approved by the Institutional Review Board (IRB) of Beykent University and conducted in compliance with the Declaration of Helsinki. Written informed consent was obtained from all participants before enrollment.

### Statistical analysis

Data collection was performed by a blinded assessor to ensure objective outcome measurement. Descriptive statistics, including means, standard deviations, medians, and ranges, were calculated for baseline demographic and clinical characteristics. The normality of data distribution was assessed using the Shapiro–Wilk test. Between-group comparisons were conducted using independent *t*-tests for normally distributed continuous variables and the Mann–Whitney *U* test for non-normally distributed continuous variables. Categorical variables, including gender, marital status, and occupational distribution, were analyzed using the Chi-square (*χ*^2^) test. Intragroup comparisons over time were analyzed using the Friedman test, as the data were non-normally distributed.

Statistical analyses were performed using SPSS version 25.0 (IBM Corp., Armonk, NY, USA), with significance set at *p* < 0.05. Analyses were conducted on an intention-to-treat (ITT) basis, with all randomized participants included in the final analysis. Missing data, if present, were handled using the last observation carried forward (LOCF) method to preserve the ITT principle.

## Results

The study included 66 participants, equally divided into two groups: 33 in the Pilates group and 33 in the home exercise group. The mean age of participants was 44.76 ± 5.67 years in the Pilates group and 44.18 ± 6.71 years in the home exercise group. The mean body mass index (BMI) was 25.92 ± 4.36 in the Pilates group and 27.63 ± 4.39 in the home exercise group. Gender distribution revealed that 90.9% of the Pilates group were women and 9.1% were men, compared to 78.8% women and 21.2% men in the home exercise group. Regarding marital status, 72.7% of the Pilates group were married, compared to 63.6% in the home exercise group. Occupational distribution showed that 15.2% of participants in the Pilates group were unemployed, 48.5% had desk jobs, and 36.4% were actively employed, whereas in the home exercise group, 24.2% were unemployed, 57.6% had desk jobs, and 18.2% were actively employed.

Statistical analysis revealed no significant differences between the groups in terms of age (*p* = 0.752), BMI (*p* = 0.119), gender distribution (*p* = 0.170), marital status (*p* = 0.428), or occupational distribution (*p* = 0.229). These findings indicate that the groups were comparable at baseline in terms of demographic characteristics and clinical evaluations, including pain, functional disability, and quality-of-life measures assessed through VAS, Roland-Morris, and SF-36 (Table 1).

**Table 1:** Comparison of pain, functional disability, and quality of life measures between the Pilates and home exercise groups

		Pilates	Home exercise	Between group *p* value
VAS score	***Before treatment***	5.27 ± 1.07	5.42 ± 0.90	*0.454*
	***Post-treatment***	2.48 ± 1.52	3.39 ± 1.14	0.010*
	***3-month***	2.12 ± 1.36	3.09 ± 1.10	0.002*
	*Intragroup p value*	< 0.001*	< 0.001*	
Rolland Morris	***Before treatment***	7.01 ± 3.21	7.30 ± 3.90	*0.928*
	***Post-treatment***	2.48 ± 2.24	4.06 ± 2.56	0.009*
	***3-month***	1.85 ± 1.37	3.91 ± 2.82	0.002*
	*Intragroup p value*	< 0.001*	< 0.001*	
SF-36 *Physical function*	***Before treatment***	53.64 ± 13.88	55.76 ± 13.12	*0.619*
	***Post-treatment***	82.12 ± 14.53	74.70 ± 13.23	0.031*
	***3-month***	85.45 ± 11.82	77.58 ± 14.04	0.025*
	*Intragroup p value*	< 0.001*	< 0.001*	
SF-36 *Physical role*	***Before treatment***	49.24 ± 13.24	47.73 ± 14.47	*0.646*
	***Post-treatment***	67.42 ± 21.18	65.15 ± 15.23	*0.759*
	***3-month***	70.08 ± 22.52	65.91 ± 16.32	*0.576*
	*Intragroup p value*	< 0.001*	< 0.001*	
SF-36 *Emotional role*	***Before treatment***	50.53 ± 24.84	49.51 ± 18.87	*0.548*
	***Post-treatment***	66.69 ± 22.05	57.60 ± 17.24	0.041*
	***3-month***	74.77 ± 18.68	65.68 ± 17.65	0.046*
	*Intragroup p value*	< 0.001*	< 0.001*	
SF-36 *Body pain*	***Before treatment***	51.52 ± 13.17	49.92 ± 10.92	*0.576*
	***Post-treatment***	75.08 ± 15.21	68.94 ± 13.56	*0.133*
	***3-month***	82.27 ± 16.59	75.15 ± 13.48	0.042*
	*Intragroup p value*	< 0.001*	< 0.001*	
SF-36 *Vitality*	***Before treatment***	56.52 ± 13.14	54.39 ± 14.02	*0.461*
	***Post-treatment***	70.00 ± 14.20	68.03 ± 14.25	*0.514*
	***3-month***	75.15 ± 13.20	71.39 ± 13.64	*0.142*
	*Intragroup p value*	< 0.001*	< 0.001*	
SF-36 *General health*	***Before treatment***	58.64 ± 13.77	54.55 ± 13.43	*0.145*
	***Post-treatment***	79.39 ± 14.18	70.91 ± 11.14	0.005*
	***3-month***	83.33 ± 12.03	75.45 ± 12.71	0.012*
	*Intragroup p value*	< 0.001*	< 0.001*	
SF-36 *Social functioning*	***Before treatment***	67.42 ± 13.95	65.15 ± 14.24	*0.523*
	***Post-treatment***	79.17 ± 13.13	74.240 ± 15.92	*0.345*
	***3-month***	81.44 ± 16.27	77.27 ± 16.67	*0.330*
	*Intragroup p value*	< 0.001*	< 0.001*	
SF-36 *Mental health*	***Before treatment***	65.94 ± 9.80	63.27 ± 14.71	*0.466*
	***Post-treatment***	73.21 ± 13.51	71.39 ± 13.64	*0.615*
	***3-month***	77.75 ± 10.60	76.94 ± 10.85	*0.953*
	*Intragroup p value*	< 0.001*	< 0.001*	

Intragroup comparisons were conducted using the Friedman test, while between-group comparisons were analyzed using independent *t*-tests or Mann–Whitney *U* tests, as appropriate. Asterisks (*) indicate statistical significance with *p* < 0.05

Italic values indicate statistically significant results with a *p*-value less than 0.05. Additionally, treatment time points and SF-36 subscales are also shown in italics for clarity

The results demonstrated significant improvements in both the Pilates and home exercise groups regarding pain intensity, functional disability, and quality of life in patients with subacute LBP. Pain intensity, measured using the VAS, decreased significantly within both groups over time (*p* < 0.001). However, the Pilates group experienced greater reductions in pain, with statistically significant differences observed post-treatment (*p* = 0.010) and at the 3-month follow-up (*p* = 0.002). Functional disability, assessed through the RMDQ, also showed significant improvements in both groups (*p* < 0.001). The Pilates group exhibited a greater reduction in scores, with significant differences favoring Pilates observed at both post-treatment (*p* = 0.009) and the 3-month follow-up (*p* = 0.002) (Table 1).

Quality of life, evaluated using the SF-36, improved in both groups across various domains, including physical function, emotional role, and general health (*p* < 0.001 for all intragroup comparisons). Between-group comparisons highlighted significantly greater improvements in the Pilates group for physical function (*p* = 0.031 post-treatment and *p* = 0.025 at 3 months), emotional role (*p* = 0.041 and *p* = 0.046), and general health (*p* = 0.005 and *p* = 0.012). For body pain, the Pilates group demonstrated a significant advantage over the home exercise group at the 3-month follow-up (*p* = 0.042). Other domains, such as vitality, social functioning, and mental health, improved similarly in both groups, with no significant differences observed between them (Table 1).

## Discussion

This study aimed to compare the effects of an eight-week Pilates-based rehabilitation program and a home exercise program on pain intensity, functional disability, and quality of life in individuals with subacute LBP. The results demonstrated that both interventions led to significant improvements in these outcomes; however, the Pilates group exhibited greater reductions in pain and disability and more substantial enhancements in health-related quality of life. These findings are consistent with previous research indicating that Pilates-based interventions effectively enhance core stability, postural control, and neuromuscular coordination in individuals with LBP [21, 26, 27].

Our findings align with existing evidence supporting the benefits of Pilates in improving functional outcomes and reducing pain in patients with chronic and subacute LBP [8, 11, 18, 19]. Several randomized controlled trials have similarly demonstrated that Pilates-based rehabilitation programs lead to greater improvements in pain relief, disability reduction, and physical function compared to general exercise programs [28–30]. This enhanced efficacy may be attributed to Pilates’ emphasis on core muscle activation, controlled movement, and proprioceptive training, all of which contribute to improved spinal stability and reduced mechanical stress on the lumbar spine [21, 31, 32].

Our study also aligns with research emphasizing the importance of early intervention in the subacute phase of LBP. Clinical guidelines suggest that early physical activity and rehabilitation can prevent the transition from subacute to chronic LBP [9, 14, 33]. Notably, a systematic review concluded that core stabilization exercises, such as those employed in Pilates, have superior long-term benefits compared to conventional exercise programs [18, 21, 34]. Furthermore, emerging evidence suggests that neuromuscular control and proprioceptive feedback mechanisms are significantly improved through structured Pilates interventions, reducing the risk of pain recurrence and long-term disability [21, 29].

The greater improvements observed in the Pilates group may be partially attributed to its emphasis on core stabilization, neuromuscular re-education, and movement control. Some studies suggest that individuals with LBP exhibit altered neuromuscular activation patterns, particularly in the transversus abdominis and multifidus muscles, and that exercises focusing on core stability, such as Pilates, may help improve muscle activation and coordination [7, 18, 35]. Pilates exercises emphasize segmental spinal control and proprioceptive awareness, which may help restore normal neuromuscular function and reduce pain recurrence [36, 37]. Additionally, the structured and supervised nature of the Pilates sessions may have contributed to higher adherence rates and more consistent execution of exercises compared to the home exercise group [18, 38, 39]. Recent studies also highlight that the mind–body connection fostered by Pilates plays a crucial role in pain perception and functional recovery [40, 41]. The integration of controlled breathing, concentration, and precise movement patterns may activate central mechanisms responsible for pain modulation and motor learning, further enhancing the long-term efficacy of Pilates-based interventions [42, 43].

The results of this study reinforce the clinical utility of Pilates as an effective rehabilitation strategy for individuals with subacute LBP. Given its ability to enhance spinal stability, neuromuscular control, and functional mobility, Pilates may be a valuable addition to current physiotherapy protocols. Furthermore, incorporating Pilates-based interventions in early-stage rehabilitation may help prevent chronicity and reduce the economic burden associated with prolonged disability [44]. Additionally, the cost-effectiveness of Pilates compared to pharmacological and invasive treatments suggests that it could serve as a viable alternative in multidisciplinary pain management approaches [14, 18].

A major strength of this study is its randomized controlled design, which minimizes bias and strengthens causal inferences. Additionally, the use of validated outcome measures, including the RMDQ and SF-36, enhances the reliability of findings. However, several limitations must be acknowledged. First, the sample size was relatively small, which may limit the generalizability of results. Second, while the study included an eight-week follow-up, longer-term assessments would be necessary to determine the sustained benefits of Pilates versus home exercises. Future research should explore the cost-effectiveness of Pilates-based rehabilitation and investigate its long-term impact on recurrence rates and chronic pain prevention.

Moreover, the lack of individualized modifications in the home exercise group may have limited its efficacy compared to the supervised Pilates intervention. Additionally, while our sample size was sufficient for detecting primary outcomes, it may have been underpowered to detect differences in certain secondary outcomes. Another potential limitation is the reliance on self-reported measures, which may introduce response bias and affect the accuracy of pain and functional assessments. Furthermore, the absence of long-term follow-up restricts our ability to assess the sustained effects of the interventions. Future studies should explore the use of tele-rehabilitation strategies and digital feedback systems to enhance adherence and optimize outcomes in home-based exercise programs. These technologies could provide real-time guidance, progress tracking, and personalized feedback, potentially bridging the gap between supervised and unsupervised exercise interventions.

## Conclusion

In conclusion, both Pilates and home exercise programs are effective in managing subacute LBP, with Pilates yielding greater improvements in pain relief, functional disability, and quality of life. The findings support the inclusion of Pilates in physiotherapy protocols for early-stage LBP rehabilitation. Future studies should investigate the long-term benefits and economic feasibility of Pilates-based interventions in diverse populations. Furthermore, integrating digital monitoring tools and remote supervision techniques may enhance adherence and optimize treatment outcomes in home-based rehabilitation programs.

## Data Availability

The datasets used and analyzed during the current study are available from the corresponding author on reasonable request.
